# A simple, *ex vivo* phagocytosis assay of *Plasmodium vivax* merozoites by flow cytometry

**DOI:** 10.1590/0074-02760190158

**Published:** 2019-10-07

**Authors:** Elizangela Farias, Fhabiane Bezerra, Djane Clarys Baia-da-Silva, Yury Oliveira Chaves, Tatiana Bacry Cardoza, Maria Edilene Martins de Almeida, Lucas Barbosa Oliveira, Pritesh Lalwani, Patrícia Puccinelli Orlandi, Marcus Vinicius Guimaraes Lacerda, Stefanie Costa Pinto Lopes, Paulo Afonso Nogueira

**Affiliations:** 1 Universidade Federal do Amazonas, Programa de Pós-Graduação Stricto Sensu em Imunologia Básica e Aplicada, Manaus, AM, Brasil; 2 Fundação Oswaldo Cruz-Fiocruz, Instituto Leônidas e Maria Deane, Programa de Pós-Graduação Stricto Sensu em Biologia da Interação Patógeno Hospedeiro, Manaus, AM, Brasil; 3 Fundação de Medicina Tropical Dr Heitor Vieira Dourado, Manaus, AM, Brasil; 4 Fundação Oswaldo Cruz-Fiocruz, Instituto Oswaldo Cruz, Programa de Pós-Graduação Stricto Sensu em Biologia Parasitária, Rio de Janeiro, RJ, Brasil; 5 Fundação Oswaldo Cruz-Fiocruz, Instituto Oswaldo Cruz, Programa de Pós-Graduação Stricto Sensu em Biologia Celular e Molecular, Rio de Janeiro, RJ, Brasil

**Keywords:** Plasmodium vivax, merozoites, MSP1, opsonising antibodies

## Abstract

As phagocytosis is the first line of defense against malaria, we developed a phagocytosis assay with *Plasmodium vivax* (*P. vivax*) merozoites that can be applied to evaluate vaccine candidates. Briefly, after leukocyte removal with loosely packed cellulose powder in a syringe, *P. vivax* trophozoites matured to the merozoite-rich schizont stages in the presence of the E64 protease inhibitor. The Percoll gradient-enriched schizonts were chemically disrupted to release merozoites that were submitted to merozoite opsonin-dependent phagocytosis in two phagocytic lines with human and mouse antibodies against the N- and C-terminus of *P. vivax* Merozoite Surface Protein-1 (Nterm-PvMSP1 and MSP1_19_). The resulting assay is simple and efficient for use as a routine phagocytic assay for the evaluation of merozoite stage vaccine candidates.

Based on *Plasmodium falciparum* studies, merozoite opsonisation appears to be correlated to immunity against malaria, and such merozoite phagocytosis assays could be useful to aid preclinical vaccine development and evaluate vaccine clinical trials.^1^ ^,^ ^2^ ^,^ ^3^ Merozoite phagocytosis has never been evaluated in *P. vivax*, hence, we adapted standardised protocols to develop a merozoite phagocytosis assay with saponin-treated *P. vivax* schizonts concentrated from clinical isolates, the flow cytometry was a useful tool for studying phagocytic uptake of blood stages.^4^ ^,^ ^5^ ^,^ ^6^ The resulting assay is a simple to evaluate opsonising antibodies from malaria vaccine candidates.

Two *Pv*MSP1 recombinant proteins (Nterm-PvMSP1 and MSP1_19_) were purified on glutathione-Sepharose 4B columns (Amersham-GE Healthcare Life Sciences, Sinapse Biotecnologia, São Paulo, Brazil) as previously described as GST tagged proteins.^7^ ^,^ ^8^ ^,^ ^9^ Each protein was used to obtain mouse-immunised sera and specific, purified human IgG antibodies from malaria-infected individuals by immunoprecipitation.

For murine-immunised sera, eight-week-old female Balb/c mice (n = 3) were intraperitoneally immunised with 50 µg protein of Nterm-PvMSP1, MSP1_19_ or Glutathione S-transferases (GST) alone dissolved in 0.1 mL phosphate-buffered saline (PBS) emulsified in 1:1 ratio with complete Freund’s adjuvant, and subsequent immunisations were administered in incomplete Freund’s adjuvant at intervals of 20 days. Control mice received PBS plus adjuvant. Mouse immunised sera were quantified by enzyme-linked immunosorbent assay (ELISA). All immunisation procedures minimised discomfort to the nonhuman animals. All procedures were approved by CEUA-INPA 03/2015. Sera from mice immunised with GST were used as controls. For human antibodies, each proteins was cross-linked to carbon nanotubes (CNTs). Briefly, activated CNTs with five µM EDAC followed by 10 mM NHS in pH 7.0 phosphate buffer were linked in ration of 100 μg of protein to 500 mg of CNTs for 30 min, blocked with bovine serum albumin (BSA) and stored at 4ºC in the presence of sodium azide until use. We used human plasma from malaria-exposed individuals whose anti*-*Nterm-PvMSP1 and anti*-*MSP1_19_ antibodies were previously identified.^9^ Pooled human plasma samples were added, incubated for 30 min under agitation at room temperature, and washed with six cycles of washing and centrifugation. For the elution of the specific anti*-*Nterm-PvMSP1 and anti*-*MSP1_19_ antibodies, 0.1 M glycine-HCl (pH 3.0) was added and coupled CNTs were centrifuged for 12,000 × g for 5 min at room temperature. To minimise acid degradation, the eluate was recovered and transferred to tubes containing 1/10th volume of 1 M Tris-HCl, pH 9.0. The reactivity of the eluted human antibodies and mouse immunised sera against anti-N-term PvMSP1 and anti-MSP1_19_ were tested using enzymatic immune assays with the respective proteins. The best opsonising concentration of antibodies was verified in cytometry with ethidium bromide labelled merozoites, the 0.5 µg/mL for purified human antibodies and 1:50 dilution of mouse immunised.

As sources of merozoites for the phagocytosis assays, five human malaria blood samples were collected from adult malaria patients at *Fundação de Medicina Tropical Dr Heitor Vieira Dourado*, FMT-HVD, for which consent forms were approved and five milliliters of peripheral blood was collected from infected patients (approval number CAAE 42021515.0.3001.0005-FMT-HVD). White blood cells were removed from the blood using a CF11 cellulose (Sigma, Brazil) column.^10^ Briefly, 50% hematocrit red blood cells were then passed through a 10 mL syringe containing five cm^3^ of loosely packed CF11-cellulose powder (Sigma, Brazil) at the bottom that was pre-sterilised by ultraviolet light. Then, *P. vivax* trophozoites were matured in 20% hematocrit in 7.5% glucose McCoy medium supplemented with 10% AB+ serum at 5% O_2_, 5% CO_2_ and 90% N_2_ until the beginning of schizogony, according to previous study.^11^ After 24-30 h of culture, parasite-infected erythrocytes were treated with trans-epoxysuccinyl-L-leucylamido (4-guanidino) butane (E64), a cysteine protease inhibitor, to ensure a maximum output of merozoite-rich schizonts, with some modifications.^12^ E64 ensured that the schizonts were fully mature after 46 h of culture and osmotically ruptured schizonts to release fully formed merozoites. The Percoll gradient confirmed the full schizogony of schizonts containing uninucleated, membrane-enclosed merozoites (Fig. 1A). The inset in this picture shows fully formed merozoites obtained after osmotic rupture. The integrity and full morphology of merozoites were verified with an immunofluorescence assay (IFA). Free merozoites and ruptured schizonts were incubated with mouse anti-N-term PvMSP1 and anti-MSP1_19_ antibodies in BSA-phosphate buffer in 1.5 mL micro tubes for 30 min at room temperature and revealed with Alexa Fluor-488 conjugated anti-mouse antibodies and DAPI were incubated for 30 min at room temperature. The images were obtained with a 100x magnitude lens using an Imaging System (EVOS-FL Color Imaging System, Thermo Fisher, Brazil). Despite the fragility of the parasites, anti-Nterm-PvMSP1 antibodies confirmed the expression of MSP1 in DAPI-labeled scattered schizonts (Fig. 1A). Free merozoites did not have damage to their surface coating after osmotic shock and repeated washings with saponin, as revealed by anti-MSP1_19_ opsonising antibodies (Fig. 1B), whereas the 19-kDa fragment (MSP1_19_) remains attached to the merozoite surface through its glycosylphosphatidylinositol anchor.^1^ ^,^ ^13^ ^,^ ^14^

**Fig. 1:**
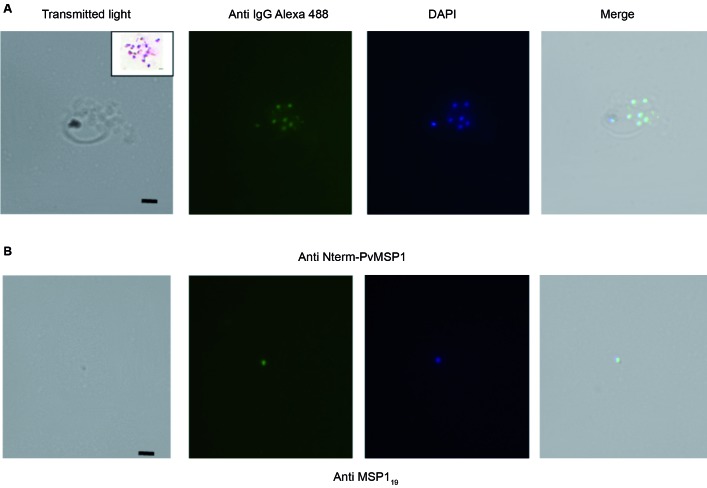
the integrity and full morphology of *Plasmodium vivax* merozoites and assessment of opsonising antibodies were verified with an immunofluorescence assay (IFA). Blood film of parasite-infected erythrocytes treated with trans-epoxy succinyl-L-leucylamido (4-guanidino) butane (E64), a cysteine protease inhibitor, that ensured a maximum output of merozoite-rich schizonts. Schizonts were osmotically ruptured with 0.1 % saponin to release fully formed and homogeneous merozoites. The integrity of the merozoite membrane was assessed with an IFA with immunised mouse sera against the Nterm-PvMSP1 and MSP1_19_ antibodies. (A) The anti-Nterm PvMSP1 antibody revealed fully membrane enclosed merozoites. The inset in this picture shows fully formed merozoites obtained after osmotic rupture. The surface localisation of the N-terminal PvMSP1 antigen is shown in mature schizonts stained with Alexa-488, mouse anti-IgG secondary antibodies, and the nucleus is stained with DAPI (panels in order: transmission light bright field, Alexa 488; DAPI, and merge). (B) The opsonising merozoite with the anti-MSP1_19_ antibodies revealed that the surface coating did not cause damage. Bar = 1 µM.

For phagocytosis assays, merozoites were labeled with 1/1,000 DNA-specific dye SYBR Green solution (Thermo Fisher) and pre-opsonised with antibodies. The merozoite-containing pellets were washed once due to the fragility of the parasites and suspended in DMEM-HAM-F12 to prepare a suspension of 10^6^ merozoites/mL. Fifty microliters of free merozoites (~5x10^4^ free merozoites) and fifty microliters of murine macrophage cell line J774 (J774 cells) and human macrophage cell line THP1 (THP-1 cells) were incubated at a 1:1 ratio in round bottom 96-well polystyrene microwell plates for 1-h incubation at 37ºC in a 5% CO_2_ atmosphere.^13^ Each condition was performed in triplicate. After incubation, the cell suspensions were washed at 200 × g for eight minutes at room temperature to remove free parasites. The pellet was suspended and fixed in 2% paraformaldehyde (PFA) and stored in the dark at 4ºC for a maximum of 24 h prior to measurement using a FACSCanto II with red-blue lasers (BD Bioscience).^15^

To optimise the functional phagocytosis assays, SYBR-labeled merozoites and phagocytic cell lines free of merozoites were acquired individually and plotted on the forward versus side scatter (FSC *vs.* SSC) axis, respectively (Fig. 2A). We distinguished merozoite and merozoite-free phagocytic cells by a merge between both gates served to define a “phagocytic cell gate”. Dot plot charts defined in the FSC versus FL-1 axis compared phagocytosis-positive gates of pre-opsonised merozoites with anti-Nterm-PvMSP1, anti-MSP1_19_and anti-GST antibodies, or non-opsonised merozoites (Fig. 2B).

**Fig. 2:**
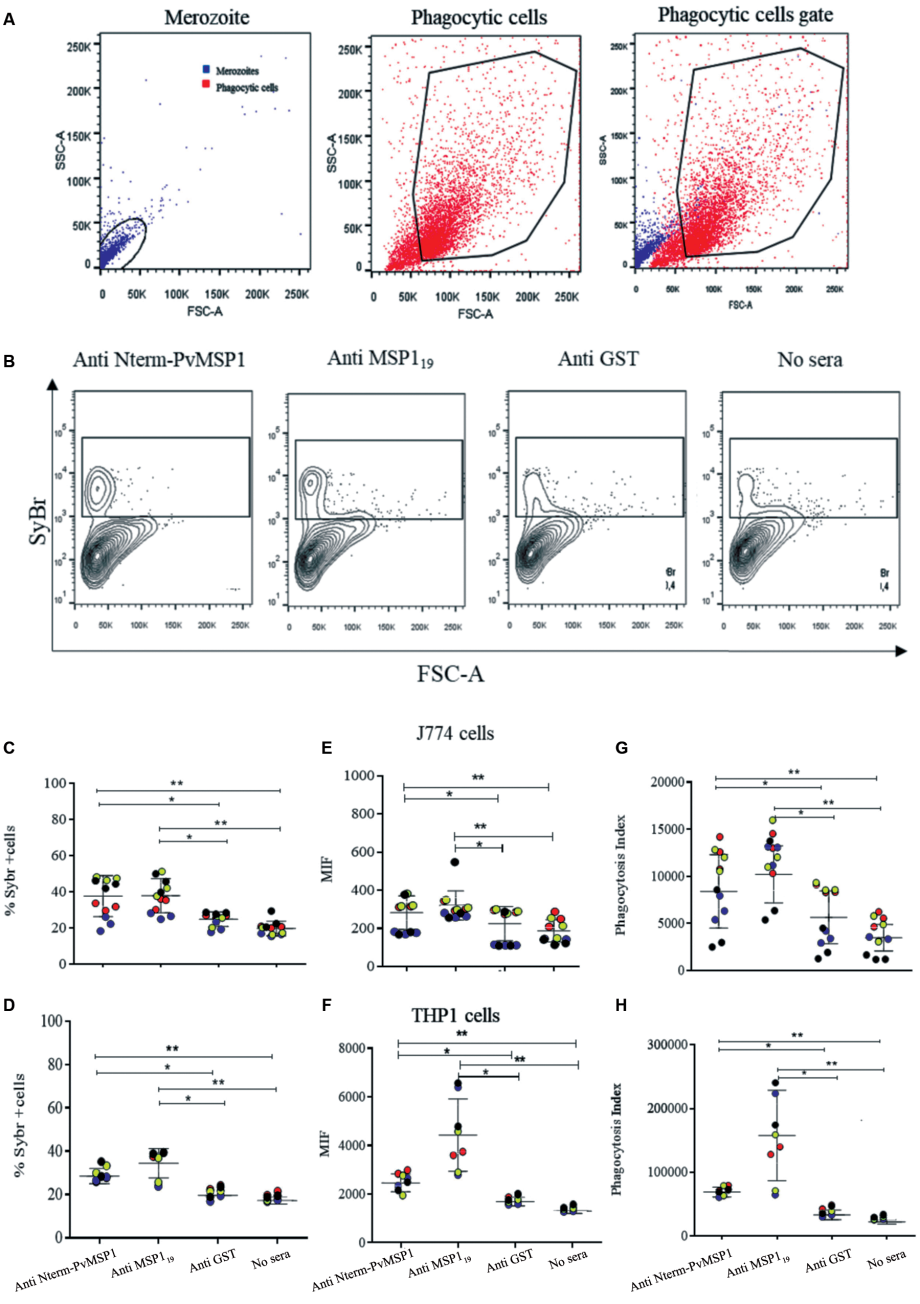
optimisation of the phagocytic cell line to target merozoites to evaluate the opsonising abilities of specific antibodies. (A) The suspension of merozoites was acquired and plotted on the FSC *vs.* SSC axis (left panel). A suspension of merozoite-free phagocytic cell lines was also plotted in the FSC vs. SSC axis (middle panel). A merge between merozoite and phagocytic cell charts served to define a “phagocytic cell gate” (right panel). (B) Contour plot charts show phagocytosis-positive gates of SYBR-labeled merozoites pre-opsonised with immunised sera; respectively anti-N-term-PvMSP1, anti-MSP1_19_ anti-GST mouse immunised sera, and no sera, measurement using a FACSCanto II with red-blue lasers (BD Bioscience). (C-H) The opsonisation-dependent merozoite phagocytosis of anti-Nterm-PvMSP1 and anti-MSP1_19_ were assessed in the murine J774 and THP-1 phagocytic cell lines. For murine J774 line, samples were tested in triplicate while with THP-1 they were performed in duplicate. For mouse antibodies, a 1:50 serum dilution in Phosphate buffered salt (PBS) of immunised sera with Nterm-PvMSP1, MSP1_19_, and GST. The PBS was used as no sera control. For purified, human IgG antibodies, a 0.5 μg/mL of purified human IgG against Nterm-PvMSP1 and MSP1_19_, and normal human IgG diluted in PBS. PBS was used as control. The results were represented individually for each sample to show variability between them. Each isolate is represented by a color that is repeated in each graph. (C-D) The percentage of SYBR-labelled merozoite phagocytising cells acquired in the phagocytosis-positive gate in relation to fifty thousand events; (C) murine J774; (D) THP-1 phagocytic cell lines. (E-F) Comparison of the median intensity fluorescence (MIF) of the SYBR-labelled merozoites of four isolates and pre-opsonised with mouse or human antibodies. (E) Murine J774; (F) THP-1 phagocytic cell lines. (G-H) The functional opsonising ability of these antibodies was assessed in phagocytosis assays with J774 and THP-1 cell lines in 96-well polystyrene round bottom plates. The phagocytosis index was standardised by multiplying the percentage of SYBR-labelled merozoite phagocytising cells by the MIF. Each condition was performed in triplicate. (G) J774 cells, (H) THP-1 cells. All data were calculated as Repeated Measures one-way ANOVA using Holm-Sidak’s multiple comparisons test. *: p < 0.05; **: p < 0.005.

The opsonisation-dependent merozoite phagocytosis of anti-Nterm-PvMSP1 and anti-MSP1_19_ were assessed in the murine J774 and THP-1 phagocytic cell lines (J774 and THP-1 cells), after 1 h at 37ºC under 5% CO_2_ (Fig. 2C-D). For murine J774 cells, samples were tested in triplicate while with THP-1 cells they were performed in duplicate. The results were represented individually for each sample to show variability between them. The percentage of SYBR+ cells were determined by the number of cells in the phagocytosis-positive gate of fifty thousand events acquired (Fig. 2C-D). The percentage of SYBR+ cells and merozoite phagocytosis levels increased significantly after opsonisation with the anti-Nterm-PvMSP1 and IgG anti-MSP1_19_ antibodies. The opsonising ability of mouse and human antibodies to anti-Nterm-PvMSP1 and anti-MSP1_19_ antibodies were evaluated by median intensity fluorescence (MIF) and calculated among events acquired in the phagocytosis-positive gate (Fig. 2E-F). To standardise merozoite phagocytosis for each sample, we created the following formula for phagocytosis index (*PI = % SYBR+ cells × MIF*). Antisera from immunised mice and purified human antibodies against Nterm-PvMSP1 and MSP1_19_ proteins were able to demonstrate the opsonising ability of the tested antibodies (Fig. 2G-H).

Based on *P. falciparum* studies, merozoite opsonisation appears to be correlated to immunity against malaria, and such merozoite phagocytosis assays could be useful to aid preclinical vaccine development and evaluate vaccine clinical trials.^3^ ^,^ ^12^ ^,^ ^13^ ^,^ ^14^ ^,^ ^16^ ^,^ ^17^ Here, merozoite phagocytosis assay with saponin-treated *P. vivax* schizonts concentrated from clinical isolates was a simple and efficient method to evaluate opsonising antibodies from malaria vaccine candidates. Because a lack of efficient and continuous *in vitro* culture systems has limited efforts to develop *P. vivax*-specific vaccines,^18^ our technique could be beneficial for evaluating *P. vivax* merozoite stage vaccine candidates.

To achieve success in merozoite phagocytosis assays, the processing of maturation *P. vivax* isolates should be rapid and efficient.^4^ ^,^ ^5^ ^,^ ^6^ After leukocyte and platelet removal, we were able to mature *P. vivax* schizonts without rupture using the E64 protease, similar to its use in *P*. *falciparum* cultures for merozoite isolation.^1^ ^,^ ^12^ ^,^ ^13^ ^,^ ^14^ ^,^ ^16^ ^,^ ^17^ Maturation was enhanced when the cultures were started when the trophozoites were mostly in the 20-24 h stage and largely dependent on leukocyte depletion, as demonstrated previously.^6^ Despite the fragility of the parasites, the integrity of the merozoite membrane was assessed with an IFA with anti-Nterm-PvMSP1 antibodies. Nterm-PvMSP1 is the major domain before the initial MSP1 proteolytic processing that releases 83, 30, and 38 kDa fragments.^7^ In addition, this is the first characterisation of anti-Nterm-PvMSP1 antibodies that confirms the expression of MSP1 in DAPI-labeled scattered schizonts. Moreover, full schizogony was also characterised with free merozoites collected from the supernatants of cultures, despite the use of E64. The immunofluorescence with the human IgG anti-MSP1_19_ antibodies confirmed the integrity of merozoites, as observed in classical studies.^1^ ^,^ ^13^ ^,^ ^14^

Our merozoite phagocytosis assay demonstrated reliability even in the presence of hemozoin, although some studies have reported that hemozoin could affect phagocyte function or generate confounding events.^1^ ^,^ ^12^ Indeed, we observed unspecific phagocytosis without antibodies; nonetheless, the phagocytosis index for the anti-Nterm-PvMSP1 and anti-MSP1_19_ antibodies was higher than in the control. Moreover, the presence of hemozoin, as a possible cause of confounding events, did not harm our merozoite phagocytosis assay, whereas an auspicious study had to modify the SSC detector to change polarised light to depolarised light to allow the detection of hemozoin.^19^ Additionally, these authors used SYBR green to distinguish Hz-containing intraerythrocytic parasites. SYBR is a cyanine dye label, and eventual confounding events could be compensated in the FL-3 channel by comparison to the normal human IgG antibodies and GST-immunised mice serum or without antibodies with sera even after compensation. Thus, due to the prominent need for physical modification, the presence of hemozoin did not harm our merozoite phagocytosis assay.

In conclusion, the impossibility of a continuous culture of *P. vivax* limited us to an *ex vivo* short-term culture with a specified endpoint: the schizont stage. We collectively standardised *P. vivax* short-term cultures and mature schizonts to be applied in investigations in endemic areas. The combination of a phagocytic assay and flow cytometry has become an efficient method for studies of malaria vaccine candidates or novel vaccine targets.
